# Real-World Impact and Educational Effectiveness of an AI-Powered Medical History-Taking System: Retrospective Propensity Score-Matched Cohort Study

**DOI:** 10.2196/89367

**Published:** 2026-02-24

**Authors:** Yang Liu, Yiying Zhu, Weishan Zhang, Xian Lu, Liping Wu, Minghui Yue, Oudong Xia, Chujun Shi

**Affiliations:** 1 Medical Simulation Center Shantou University Medical College Shantou, Guangdong China; 2 Department of Medical Physics and Informatics Shantou University Medical College Shantou, Guangdong China; 3 Shantou University Medical College Shantou, Guangdong China

**Keywords:** AI-powered medical history-taking training and evaluation system, medical history-taking, virtual patient, educational effectiveness, retrospective cohort study, propensity score matching, average treatment effect on the treated, self-regulated learning, aptitude-treatment interaction, precision education

## Abstract

**Background:**

Medical history-taking is a core clinical skill; yet, traditional teaching methods face challenges. We developed an artificial intelligence–powered medical history-taking training and evaluation system (AMTES) and established its technical feasibility as an extracurricular resource. Evidence on whether such tools improve learning outcomes when voluntarily embedded in routine curricula remains limited.

**Objective:**

This study aimed to evaluate the real-world educational effectiveness of AMTES as an opt-in extracurricular tool and examine whether learning gains vary by practice patterns and baseline academic ability.

**Methods:**

We conducted a retrospective cohort study of the 2024-2025 Diagnostics course cohort (N=478) at Shantou University Medical College, China, using total population sampling. Students were categorized as AMTES users (n=205, 42.9%; ≥1 sessions) and nonusers (n=273, 57.1%) based on their voluntary extracurricular adoption of the system during the month preceding a high-stakes final practical skills examination. To address selection bias, we performed 1:1 propensity score matching via logistic regression using age, sex, and 3 previous academic scores as covariates. The average treatment effect on the treated for final examination score (0-70) was estimated with paired *t* tests, and robustness to unobserved confounding was assessed via Rosenbaum sensitivity analysis. Among matched users, practice patterns were identified using K-means clustering on log-derived features, with cluster differences compared using Mann-Whitney *U* tests. Subsequently, we explored aptitude-treatment interaction by testing the interaction between practice intensity and baseline ability using linear and logistic regression models.

**Results:**

Propensity score matching yielded 157 matched pairs (n=314) with excellent covariate balance (|standardized mean difference|<0.1). In the matched cohort, the users outperformed nonusers by 3% (average treatment effect on the treated=2.09, 95% CI 0.75-3.42; *P=*.002). This finding was robust to weak unmeasured confounding (Rosenbaum Γ=1.23). Among users (N=157), cluster analysis of usage logs revealed a low-intensity group (74/157, 47.1%) and a high-intensity group (83/157, 52.9%). The 2 groups reflected differences in both practice quantity and quality. However, the added efforts did not translate into higher scores (mean difference=1.6 points, 95% CI −0.5 to 3.6) or excellence probability (risk difference=7.7 percentage points, 95% CI −5.0 to 20.5). Exploratory aptitude-treatment interaction analyses suggested ability-dependent effects for excellence rate (*β*_3_=1.461; *P*=.04) and marginally for final score (*β*_3_=2.58; *P*=.07), but not for pass rate (*P*=.94).

**Conclusions:**

Building upon previous technical validation, this study contributes real-world effectiveness evidence by evaluating AMTES as a voluntary extracurricular supplement within an authentic, high-baseline curriculum. Unlike previous work focusing on technical feasibility or short-term controlled trials, voluntary extracurricular AMTES use was associated with modest yet meaningful improvements in summative history-taking performance. Exploratory analyses indicated that the added value of more intensive engagement may be moderated by baseline academic ability. These findings support the scalability of artificial intelligence–enabled supplementary training and inform precision-oriented instructional design.

## Introduction

Medical history-taking is the cornerstone of clinical diagnosis and decision-making. Effective training in this skill is essential for improving diagnostic accuracy [1-3] and is a necessary prerequisite for cultivating competent physicians [4]. Consequently, how to effectively cultivate this practical skill remains a core challenge for medical educators [5]. According to Kolb’s experiential learning cycle, skill acquisition requires learners to engage in specific, feedback-driven practice activities to internalize knowledge and effectively develop skills [6]. However, medical education primarily relies on standardized patients (SPs) for this training. The high costs and limited resources associated with SPs [7] restrict the capacity to meet the demands for large-scale, high-frequency, and flexible practice opportunities required for “deliberate practice.”

With advancements in digital technologies, particularly revolutionary breakthroughs in large language models (LLMs), LLM-driven virtual patients (VPs) are viewed as a potential innovative solution to resource shortages [8-10]. VPs can function as “interactive practice objects,” allowing students to repeatedly drill scenarios and receive contextualized feedback in a safe, on-demand environment [11,12]. However, the direct application of general-purpose LLMs in medical education still faces technical limitations—such as “hallucinations” [13], reliability issues, and inadequate long-context processing capabilities—thereby undermining pedagogical accuracy and trustworthiness. Therefore, how to effectively and equitably integrate customized AI technology into medical education remains a monumental challenge [14].

To address these challenges and ensure pedagogical reliability, our research team developed the artificial intelligence–powered medical history-taking training and evaluation system (AMTES) [15]. This is a custom-designed educational platform powered by the DeepSeek LLM and intended to supplement existing teaching within a blended learning framework. The system features a multiturn dialogue module that simulates realistic, emotionally expressive patient encounters and integrates an automated assessment and feedback system based on clinical expert standards. Notably, our previous prospective multicase validation study established a “three-element” foundation for AMTES as a mature educational tool, that is (1) technical reliability (dialogue accuracy >97%; automated scoring consistency with human experts, intraclass correlation coefficient>0.92), (2) pedagogical appropriateness (training cases meticulously designed and difficulty-validated), and (3) student acceptance (87% found it helpful) [15]. This validation confirmed AMTES’s transition from being “technically functional” to “pedagogically usable.”

AMTES has been deployed as a long-term auxiliary resource for extracurricular autonomous learning in the Diagnostics course. Despite the increasing prevalence of artificial intelligence (AI) tools [12,16,17], existing research predominantly focuses on technical feasibility, dialogue quality, and assessment validity [18-20], as well as learners’ subjective experiences [21]. Consequently, high-quality evidence regarding the objective, long-term educational outcomes of AI history-taking tools in authentic, ecological educational settings remains scarce. A few studies attempting to evaluate educational effectiveness [22,23] typically use small-sample controlled trials with mandatory, prescribed-dose, short-term training. These highly controlled designs lack ecological validity, failing to reveal students’ authentic self-regulated learning (SRL) patterns during routine, voluntary use, or to reflect the long-term impact of these behaviors on subsequent summative examinations.

Therefore, it is important to focus on SRL as a core mechanism, as it has been widely recognized as a key determinant of academic achievement and clinical competence in medical education [24]. However, most quantitative SRL studies still rely on self-report questionnaires (eg, the Motivated Strategies for Learning Questionnaire), which are vulnerable to social desirability and recall bias, and can only provide static, single-point-in-time snapshots. Yet, SRL is fundamentally a dynamic, cyclical process involving forethought, performance control, and self-reflection surrounding a specific task [25]. Many such instruments were initially developed for traditional classroom settings and may not adequately capture how learners self-regulate in blended or technology-enhanced environments [26]. In light of this, recent research increasingly advocates for the use of process data and event-based measurements [25], such as log data from intelligent tutoring systems and AI-supported platforms [27], to obtain real-time, fine-grained, and objective evidence of how learners implement SRL strategies in authentic learning contexts.

Crucially, research exploring whether students with different ability levels or practice behavior patterns derive varying degrees of benefit from AI tools remains extremely limited. Educational psychology frameworks, such as the aptitude-treatment interaction (ATI) theory [28], emphasize that the impact of instructional interventions is often not uniform but varies systematically with learner characteristics [29,30]. This critical gap highlights the urgent need to investigate the heterogeneity of effects in AI-supported history-taking training across different learner groups.

This study is uniquely situated within a high-baseline training context, where participants had been receiving high-quality SP training before the introduction of AMTES. Therefore, our primary objective is to determine the marginal contribution of AMTES to existing instruction. To address the endogeneity issues inherent in such opt-in, extracurricular tools (eg, omitted variable bias) [31,32], this study used a retrospective cohort design combined with propensity score matching (PSM) to provide robust and impartial causal estimates.

Building on the successful validation of AMTES and addressing the gaps in current research, this study aims to systematically address three incremental core questions, moving from average effects to process mechanisms and heterogeneity:

- First, within the context of existing SP-assisted instruction, does the voluntary, supplementary use of AMTES yield a significant average treatment effect on the treated (ATT) on students’ practical skills?
- Second, among AMTES users, do different self-regulated practice behavior patterns (eg, low-intensity vs high-intensity) lead to different academic outcomes?
- Finally, does the effectiveness of different practice behavior patterns vary according to students’ initial aptitude, suggesting the presence of an ATI effect?

By systematically investigating these questions, this study aims to provide causal evidence for the real-world integration of customized medical LLMs, offer practical insights for instructional design, and illuminate the path toward personalized and precision medical education.

## Methods

### Study Design, Setting, and Participants

We conducted a retrospective cohort study to evaluate the real-world impact of the AMTES on student performance in an undergraduate Diagnostics course at Shantou University Medical College in the 2024-2025 academic year. This study is reported in accordance with the Journal Article Reporting Standards for quantitative research [33]. Total population sampling was used. All undergraduate medical students enrolled in the course were included (280/478, 58.6% male and 198/478, 41.4% female; mean age 20.38, SD 0.72 years). Because this was a complete-course cohort and the exposure was determined retrospectively, no a priori sample size calculation was performed.

All students, regardless of subsequent AMTES use, received the standard curriculum prescribed by the medical school, which comprised regular didactic lectures and in-person practical training sessions with SPs accompanied by individualized feedback from clinical instructors. This SP-based training constituted the primary instructional foundation for the acquisition of history-taking skills for all students.

One month before the final practical skills examination (held in early July 2025), AMTES was introduced as a voluntary extracurricular supplement. Students were free to decide whether and how often to use the system for additional practice during this 1-month period. Based on their actual usage, we retrospectively categorized students into 2 cohorts—an AMTES user group (≥1 training sessions) and a nonuser group (0 sessions).

After the course concluded, AMTES system usage logs were aggregated and linked with students’ course examination scores. This design enabled comparison of outcomes between AMTES users and nonusers and allowed us to estimate the marginal educational value of AMTES on top of an existing high-quality curriculum.

### Procedures

#### System Implementation

**Figure 1:**
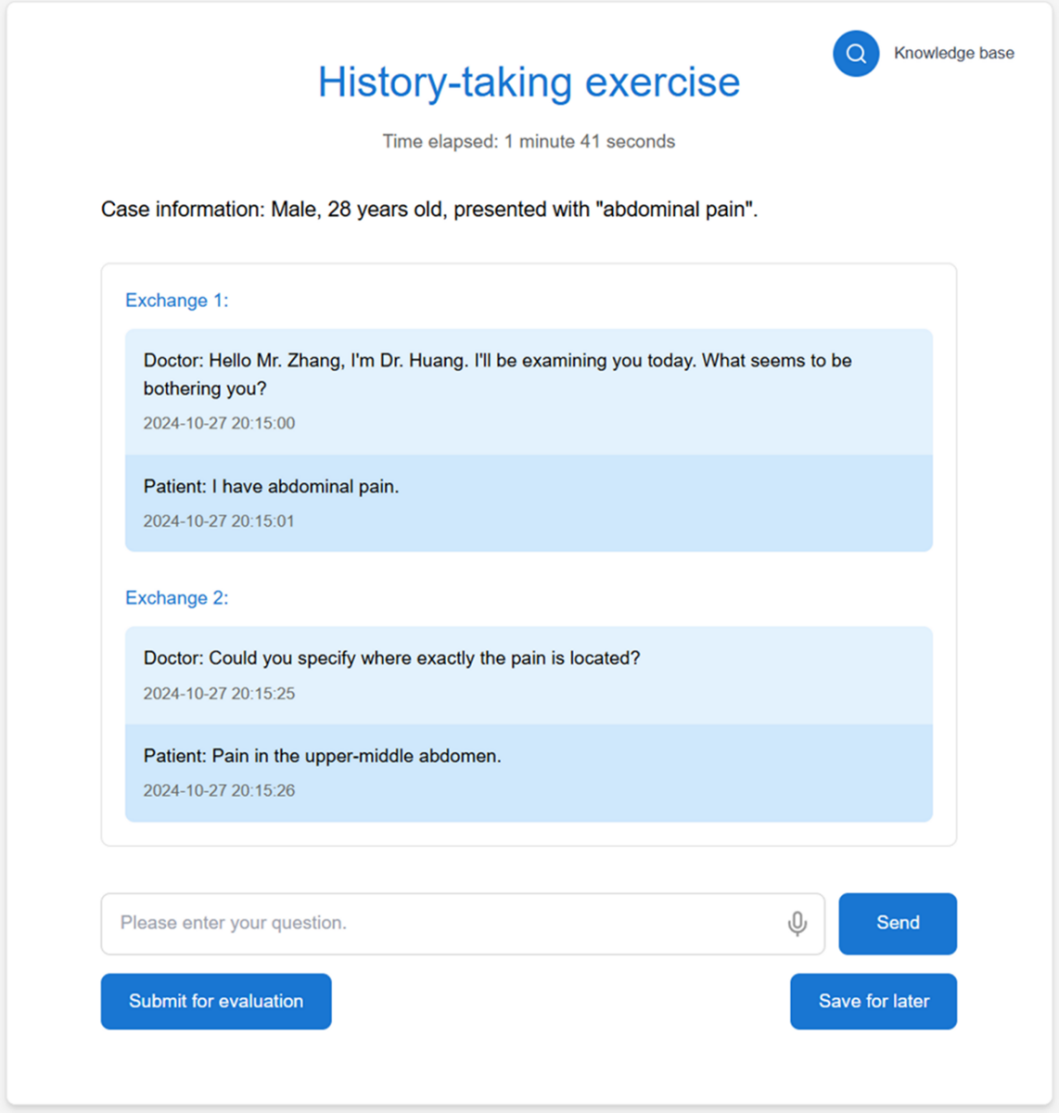
Screenshot of history-taking interface where students could conduct multiturn consultations.

During the month preceding the final practical skills examination, AMTES was made available to all students in the Diagnostics course as a voluntary extracurricular resource. The system provides a web-based interface in which students conduct multiturn consultations with a VP using either typed or voice input (Figure 1). During each simulated consultation, AMTES generated patient responses in real time and recorded the full question-answer dialogue.

**Figure 2:**
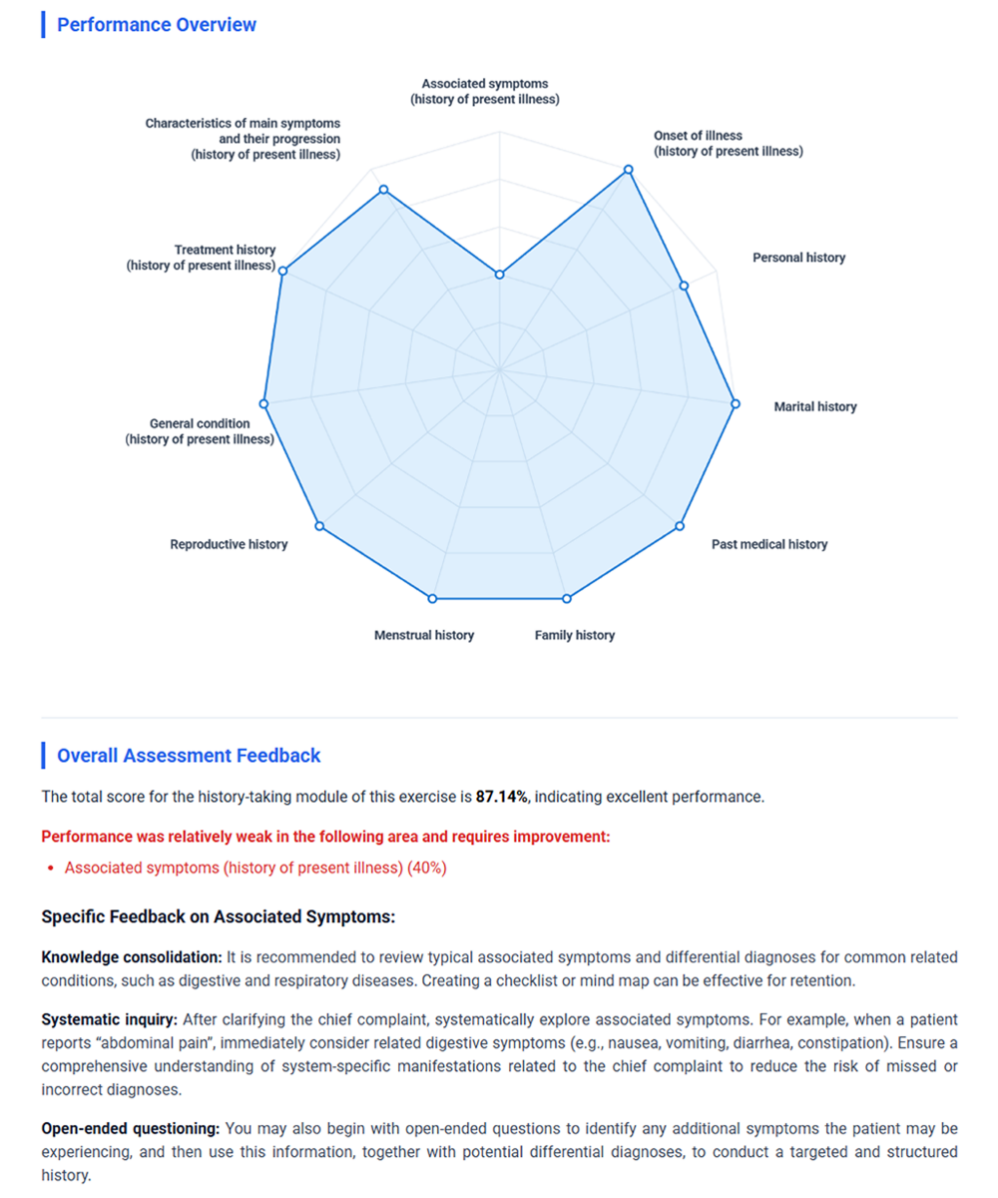
Example of an automated assessment feedback report from the artificial intelligence–powered medical history-taking training and evaluation system.

At the end of each session, students received an immediate structured feedback report summarizing their overall performance, subscores across key skill dimensions, missed critical items, and suggestions for improvement (Figure 2). These automated reports were intended to emulate an instructor’s critique and provide timely guidance for self-reflection and adjustment.

AMTES offered 3 validated training cases of increasing difficulty, covering different chief complaints and organ systems, that are cough and sputum (simple), abdominal pain (moderate), and urinary frequency (complex). The underlying diagnoses and core symptoms of these training cases were designed not to overlap with those of the final practical skills examination case, thereby reducing the risk of direct teaching to the test. Students could use AMTES at any time and as often as they wished during the 1-month period. In practice, 205 (42.9%) students completed at least 1 AMTES session (user group), whereas 273 (57.1%) did not (nonuser group).

#### Final Practical Skills Examination and Scoring

The summative assessment consisted of a 20-minute one-on-one history-taking consultation with an SP. An assessor-blinded design was implemented to ensure objectivity, whereby examiners were blinded to students’ AMTES usage status. Furthermore, given the retrospective nature of this study, students were unaware at the time of examination that their AMTES usage data would be analyzed in subsequent research, thereby minimizing potential behavioral modifications due to research awareness (Hawthorne effect). All participating SPs underwent rigorous pre-examination training on the case script and performance baseline standards to ensure consistency and standardization. Examiners also received standardized training to ensure uniform application of the scoring criteria.

The scoring rubric was developed with reference to the National Clinical Practitioner Licensing Examination standards of China, with a maximum score of 70 points. The rubric covered 6 key domains—chief complaint, history of present illness, past medical history, personal history, marital and reproductive history, and family history. Points were allocated according to clinical importance, with each scoring item corresponding to a single, clearly defined evaluation criterion. To maximize objectivity and minimize examiner subjectivity, a dichotomous scoring method was used: students received full points for an item if the required information was successfully elicited, and zero points otherwise.

According to institutional academic regulations, performance grades were classified as excellent (≥85%), pass (60%-84%), and fail (<60%). Since the history-taking component in this study had a maximum raw score of 70 points, we applied an equivalence ratio of 0.7 to convert these institutional thresholds. Specifically, we defined ≥42 points (ie, 60% of the maximum score) as the “pass” threshold and ≥59.5 points (ie, 85% of the maximum score) as the “excellence” threshold.

#### Data Collection, Measures, and Covariates

##### Overview

All data used in this study were obtained from anonymized AMTES usage logs and official academic records, capturing both students’ actual learning behaviors and their performance outcomes in an authentic educational context. We considered four types of variables, that is, baseline covariates, intervention variables, outcome variables, and practice behavior logs.

##### Baseline Covariates

We selected 5 baseline covariates as predictors for the propensity score model. These included demographic characteristics (age and sex) and 3 academic performance metrics obtained before the AMTES period—the current course midterm examination score, previous semester theory examination score, and previous semester practical skills examination score, representing students’ baseline academic aptitude. These academic metrics were chosen because previous performance is a robust proxy for underlying attributes, such as motivation, self-discipline, and previous knowledge [34,35]. Additionally, age and sex were incorporated as key individual-difference variables highlighted in the unified theory of acceptance and use of technology 2 framework, which identifies them as influential factors in technology adoption and usage behavior [36].

##### Intervention Variable

A binary indicator of AMTES use during the 1-month window, defining a user group (≥1 training sessions) and a nonuser group (0 sessions).

##### Outcome Variables

The primary outcome was the total score (0-70 points) on the final medical history-taking skills examination. Secondary outcomes were 2 dichotomous indicators derived from this score, that is, excellence (score ≥59.5) and pass (score ≥42), based on institutional grading standards.

##### Practice Behavior Logs

For AMTES users, we extracted behavioral metrics from the system logs, including the total number of sessions, the number of unique cases practiced, session-level AMTES scores, and dialogue turns. These log-based indicators were used as behavioral proxies of students’ SRL processes when engaging with AMTES.

### Statistical Analysis

#### Overview

All statistical analyses were conducted using *R* (version 4.2.0; *R* Foundation for Statistical Computing) and Python (version 3.10; Python Software Foundation). A 2-sided significance level of α=.05 was used. Since all study data, including digital trace logs and academic records, were automatically captured and integrated, no missing data were identified for the baseline covariates or outcome variables analyzed in this cohort. Continuous variables were summarized as mean (SD) or median (IQR), and categorical variables as counts and percentages.

#### PSM Analysis

To mitigate selection bias arising from voluntary AMTES use, we applied PSM to construct comparable cohorts of users and nonusers. Propensity scores were estimated using a logistic regression model with AMTES use (yes or no) as the dependent variable, conditioned on the 5 aforementioned baseline covariates. We performed 1:1 nearest-neighbor matching without replacement, using a caliper of 0.2 times the SD of the logit of the propensity score. Additionally, exact matching on sex was imposed to ensure sex comparability within each matched pair. Covariate balance was assessed using standardized mean difference (SMD), with |SMD|<0.1 indicating adequate balance [37]. A Love plot was generated to visually inspect covariate balance.

Within the matched sample, we first examined the normality of within-pair differences in final practical skills examination scores using the Shapiro-Wilk test, which did not indicate substantial deviations from normality (Multimedia Appendix 1). On this basis, we estimated the ATT by comparing final practical skills examination scores between AMTES users and matched nonusers using paired *t* tests, reporting mean differences with 95% CIs. To assess the robustness of the findings to potential unmeasured confounding, we conducted a Rosenbaum sensitivity analysis [38] and reported the Γ value at which the ATT would no longer be statistically significant.

#### Practice Behavior Pattern Clustering

To identify distinct SRL-related practice patterns among AMTES users in the matched cohort (n=157), we performed an unsupervised clustering analysis based on log-derived features. From the practice logs, we initially extracted 11 candidate features across 4 conceptual dimensions—engagement and coverage, practice strategy, platform performance, and interaction dynamics. Before clustering, the positively skewed metric (total_sessions) was log-transformed, and all continuous variables were centered and scaled. To reduce redundancy, we examined pairwise Spearman rank correlations among candidate features. For highly correlated pairs (*|ρ|*≥0.80), we retained the feature with greater interpretability and conceptual relevance. Variance inflation factors computed on the reduced feature set confirmed acceptable multicollinearity (all variance inflation factors<5). The final clustering used 5 retained features (Table 1). Full details of feature construction and selection appear in Multimedia Appendix 2.

We used the K-means algorithm to cluster students based on these features. The optimal number of clusters was determined to be 2 (K=2) using the silhouette coefficients. To ensure stability, K-means was run with multiple random initializations, which yielded consistent cluster assignments (Multimedia Appendix 3). Each student was subsequently assigned to 1 of 2 clusters for subsequent comparisons. Given the nonnormal distribution of log-derived performance metrics, we used the Mann-Whitney *U* test (2-sided) to compare cluster characteristics.

**Table 1:** Log-derived features used for clustering analysis among matched artificial intelligence–powered medical history-taking training and evaluation system users (n=157).

Feature^a^	Interpretation
Sessions_total^b^	Total number of AMTES^c^ sessions completed
Unique_cases	Number of distinct cases practiced
Avg_score	Mean session score across all sessions
Avg_turns	Average number of dialogue turns per session
Turns_per_min	Overall dialogue turns per minute

^a^All features were standardized (*z* scores) before clustering.

^b^Skewed variable was transformed using a log1p transformation (log[x+1]).

^c^AMTES: artificial intelligence–powered medical history-taking training and evaluation system.

#### Exploratory Analysis of Heterogeneous Effects

To address our third research question, we conducted an exploratory analysis grounded in the ATI framework, examining whether the effectiveness of different AMTES practice patterns varied by baseline academic ability. Within the AMTES user group, we constructed a standardized composite baseline ability score by averaging *z* scores of midterm, previous theory, and previous practical skills examination scores. We then fitted 3 regression models with identical predictors—cluster assignment (practice pattern), the composite baseline ability score, and their interaction term. A linear regression model was used for the continuous final examination score, and 2 logistic regression models were used for the binary outcomes of pass (score ≥42 vs <42) and excellence (score ≥59.5 vs <59.5).

Interaction terms were tested using Wald tests. To aid interpretation and generate hypotheses for future research, we computed and plotted marginal effects of being in the high-intensity versus low-intensity group at the 25th, 50th, and 75th percentiles of the baseline ability score. For the linear model, marginal effects were expressed as differences in predicted final practical skills examination scores; for the logistic models, they were expressed as risk differences in pass and excellence probabilities, each with 95% CIs.

### Ethical Considerations

This study received ethical approval from the Ethics Committee of Shantou University Medical College (approval SUMC-2025-028). All procedures were conducted in accordance with the principles of the Declaration of Helsinki and complied with relevant Chinese laws and institutional ethical standards. Before participation, all participants provided written informed consent. To ensure the privacy and confidentiality of participants, all data were anonymized before analysis and used solely for academic research purposes. No financial compensation was provided to participants for their involvement in this study. All images presented in this paper contain only simulated demonstration data and do not display any identifiable information of actual participants. No identifiable images of individual participants appear in the manuscript or supplementary materials.

## Results

### ATT of AMTES on Final Practical Skills Examination Scores

#### Baseline Characteristics

**Figure 3:**
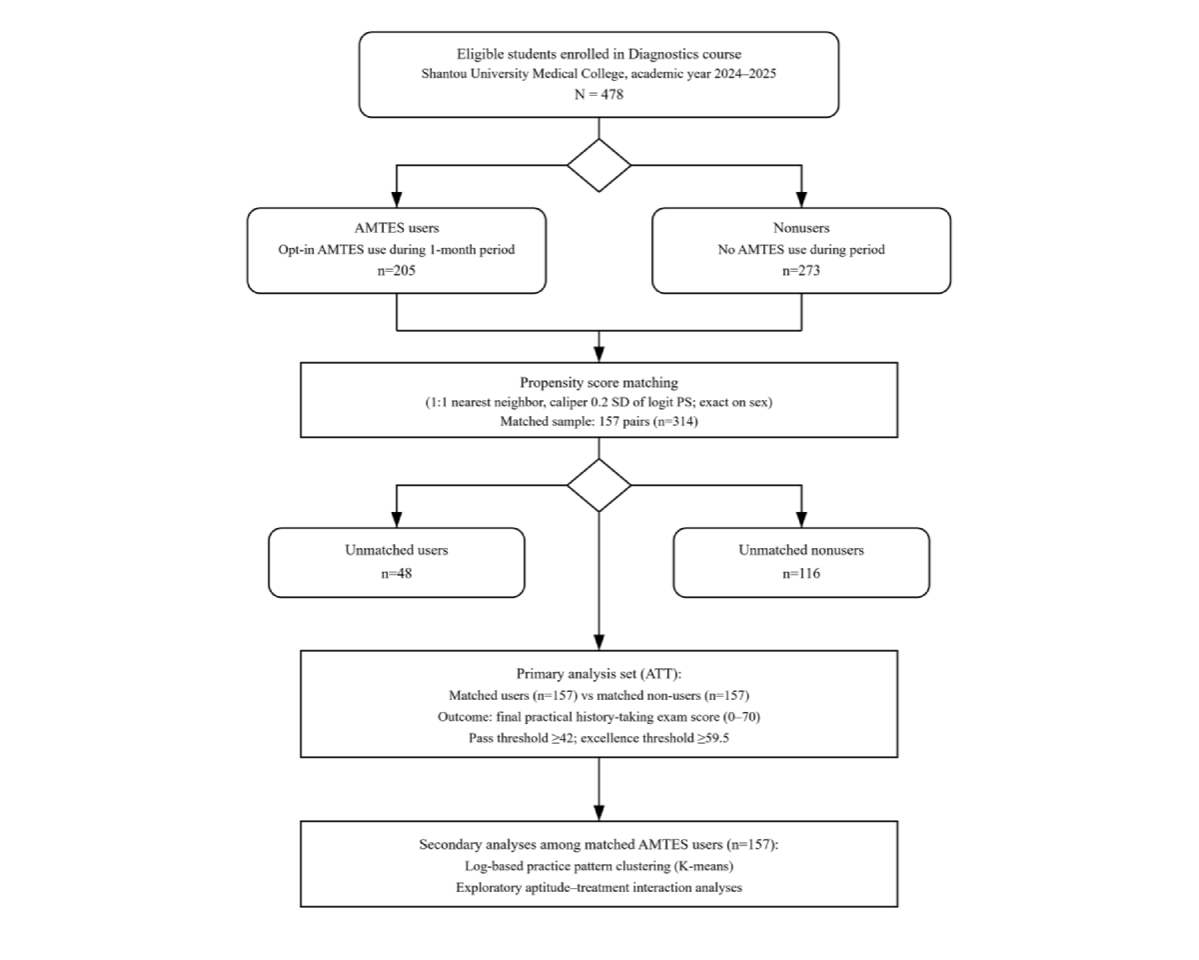
Study flow diagram of participant selection and propensity score matching. AMTES: artificial intelligence–powered medical history-taking training and evaluation system; ATT: average treatment effect on the treated.

Overall, 478 undergraduate students were included in the initial cohort, comprising 205 AMTES users and 273 nonusers (Figure 3). Before matching, significant selection bias was evident. The user group demonstrated a stronger academic baseline (absolute SMD far exceeding the |0.1| threshold for all academic covariates) and differed in gender distribution (|SMD|=0.21) compared with the nonuser group. The propensity score model showed moderate discriminatory ability (area under the receiver operating characteristic curve=0.690; Multimedia Appendix 4).

**Figure 4:**
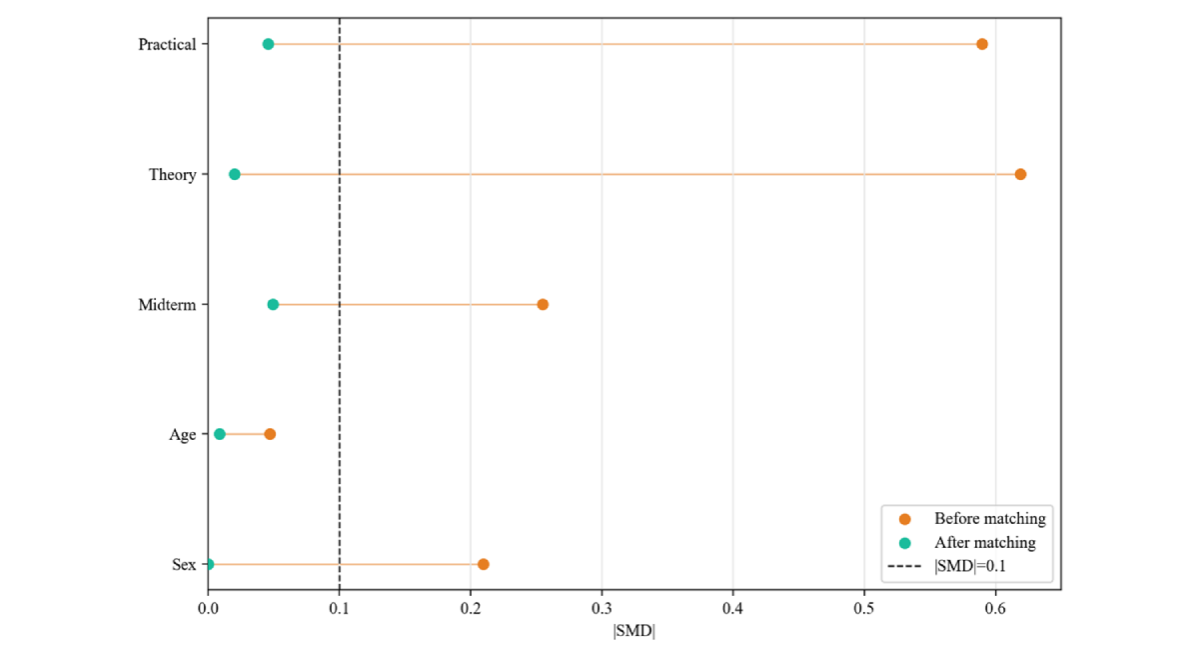
Love Plot of baseline covariates before and after propensity score matching (N=478). SMD: standardized mean difference.

After 1:1 nearest-neighbor matching was performed, 157 matched pairs (n=314) were retained (Figure 3). All covariates achieved excellent balance (|SMD|<0.1), and the PS model’s area under the receiver operating characteristic curve was correspondingly reduced to a chance level of 0.521 (Figure 4; Table 2; Multimedia Appendix 4). These results indicated that PSM successfully controlled for observable confounding variables, creating comparable cohorts for evaluating the effect of AMTES.

**Table 2:** Comparison of covariate balance metrics before and after matching (N=478).

Metric			Before matching												After matching								
			Treated		Control		SMD^a,b^			AUROC^c^			Treated				Control		SMD^b^			AUROC	
Total (N=478), n (%)			205 (42.9)		273 (57.1)		—^d^			—			157 (76.6)^e^				157 (57.5)^f^		—			—	
**Sex, n (%)^g^**							0.21			—									0			—	
	Male	108 (52.7)		172 (63)								92 (58.6)				92 (58.6)							
	Female	97 (47.3)		101 (37)								65 (41.4)				65 (41.4)							
Age (y), mean (SD)			20.380 (0.722)		20.414 (0.702)		0.047			—			20.408 (0.742)				20.401 (0.724)		0.009			—	
Midterm examination, mean (SD)			26.361 (2.652)		25.571 (3.491)		0.255			—			26.121 (2.809)				26.248 (2.369)		0.049			—	
Theory examination, mean (SD)			72.185 (11.472)		64.385 (13.636)		0.619			—			68.873 (10.906)				69.096 (11.345)		0.02			—	
Practical skills examination, mean (SD)			76.487 (9.886)		70.083 (11.752)		0.590			—			73.604 (9.2)				74.036 (9.762)		0.045			—	
Final examination, mean (SD)			54.311 (6.427)		50.738 (7.239)		—			—			53.788 (6.591)				51.701 (6.577)		—			—	
Overall			—		—		—			0.69			—				—		—			0.521	

^a^SMD: standardized mean difference.

^b^Covariates were considered well-balanced if the absolute standardized mean difference was <0.1.

^c^AUROC: area under the receiver operating characteristic curve.

^d^Not applicable.

^e^Retention was 76.6% for the treated group.

^f^Retention was 57.5% for the control group.

^g^For propensity score estimation, sex was coded as male=1 and female=0.

#### Final Practical Skills Examination Scores

Analysis of the 157 matched pairs revealed a significant, positive effect of AMTES on final practical skills examination scores. The user group achieved a higher mean score than the nonuser group (mean 53.788, SD 6.591 vs mean 51.701, SD 6.577). The effect was 2.09 points (95% CI 0.75-3.42; *P*=.002). Given a maximum score of 70, this was equivalent to an increase of approximately 3% of the total score, suggesting a practically meaningful impact.

A Rosenbaum sensitivity analysis showed that this conclusion was robust to unobserved confounding of weak strength. An unobserved confounder would need to increase the odds of a student using AMTES by a factor of approximately 1.23 to render the difference nonsignificant (*P*>.05).

### Practice Behavior Clustering Results

K-means clustering (K=2) identified 2 distinct practice patterns among the 157 AMTES users based on a multidimensional construct of usage intensity, which incorporated practice frequency, case coverage, average session score, and interaction intensity in the matched cohort (Table 3). Cluster 1 (n=74, 47.1%) was characterized by lower practice frequency and narrower case coverage, referred to as a low-intensity group. In contrast, cluster 2 (n=83, 52.9%) demonstrated substantially higher activity across all dimensions—practice frequency, case coverage, and interaction intensity (all *P*<.001; Table 3), referred to as a high-intensity group. Average score differences were smaller but significant (*P*=.04). These patterns reflected differences in both practice quantity and quality.

While the 2 user groups demonstrated significantly different practice behaviors, the added effort did not yield a significant performance advantage. Both groups achieved high pass rates approaching 100% (high-intensity 94% vs low-intensity 97.3%), leaving little room for improvement at this threshold. The potential benefits of higher practice intensity were more evident in the excellence rate (25.3% vs 17.6%) and mean score (54.52, SD 6.4 vs 52.97, SD 6.7; Cohen *d*=0.24), although these differences remained nonsignificant (Table 4).

**Table 3:** Practice behavior characteristics of the two practice intensity clusters among matched artificial intelligence–powered medical history-taking training and evaluation system users (n=157).

Performance feature	Low-intensity group, mean (SD)	High-intensity group, mean (SD)	*P* value
Sessions_total	1.243 (0.463)	3.205 (1.166)	<.001
Unique_cases	1.135 (0.344)	2.590 (0.495)	<.001
Avg_score	43.677 (10.752)	47.603 (5.351)	.04
Avg_turns	67.029 (28.849)	89.379 (23.240)	<.001
Turns_per_min	2.684 (0.836)	3.462 (0.872)	<.001

**Table 4:** Comparison of final practical skills examination scores between high- and low-intensity practice groups among matched artificial intelligence–powered medical history-taking training and evaluation system users (n=157).

Outcome metric	Low-intensity cluster (n=74)	High-intensity cluster (n=83)	Risk difference, percentage points (95% CI)	Relative risk (95% CI)	Mean difference (95% CI)	Cohen *d* (95% CI)
Excellence rate (≥59.5)	n=13 (17.6%)	n=21 (25.3%)	7.7 (−5.0 to 20.5)	1.44 (0.78 to 2.67)	—^a^	—
Pass rate (≥42)	n=72 (97.3%)	n=78 (94%)	−3.3 (−9.6 to 3.0)	0.97 (0.90 to 1.03)	—	—
Total score (0-70)	Mean 52.97 (SD 6.7)	Mean 54.52 (SD 6.4)	—	—	1.6 (−0.5 to 3.6)	0.24 (−0.08 to 0.55)

^a^Not applicable.

### Exploratory Analysis of Heterogeneous Effects: Moderating Role of Baseline Ability

Regression models examining ATI suggested that practice intensity effects may vary by baseline ability for excellence rate (*β*_3_=1.461; *P*=.04) and final score (*β*_3_=2.58; *P*=.07), but not for pass rate (*P*=.94).

**Figure 5:**
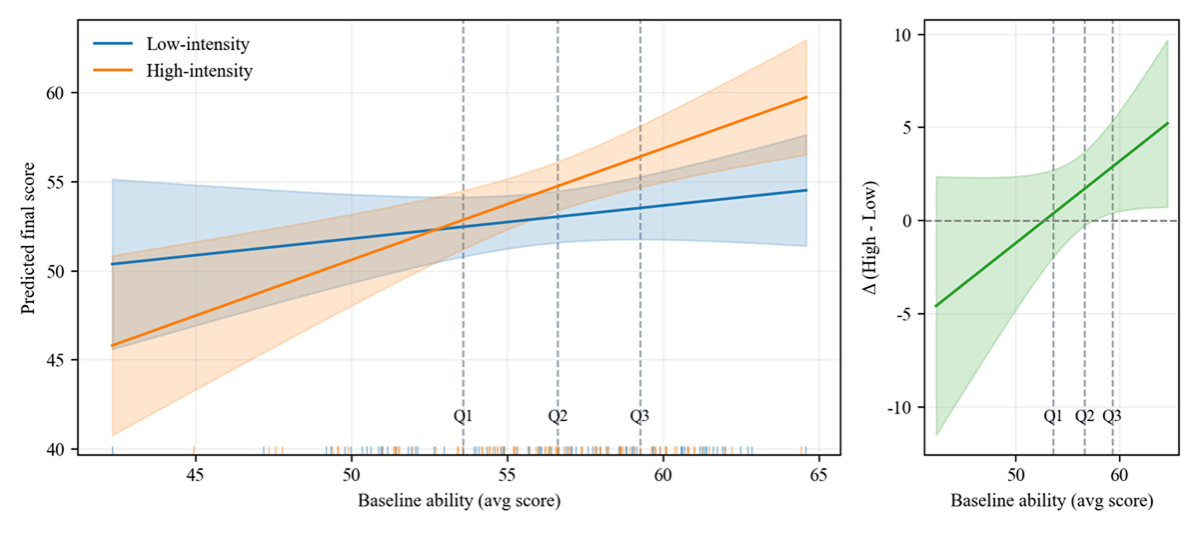
Predicted final examination scores (0-70) by practice intensity and baseline ability index among matched artificial intelligence–powered medical history-taking training and evaluation system users (n=157). Baseline ability was defined as a composite z score: baseline_z=mean[z(practical), z(theory), z(midterm)]. For interpretability, baseline_z was mapped to an “Avg score” scale: Avg_score=mean(raw_avg)+baseline_z×SD(raw_avg), where raw_avg=(practical+theory+midterm)/3; quartile reference points: Q1=−0.45 (Avg_score=53.58 approximately), Q2=0.07 (56.62 approximately), Q3=0.52 (59.26 approximately); Rug marks show baseline ability distribution by cluster. avg: average.

**Figure 6:**
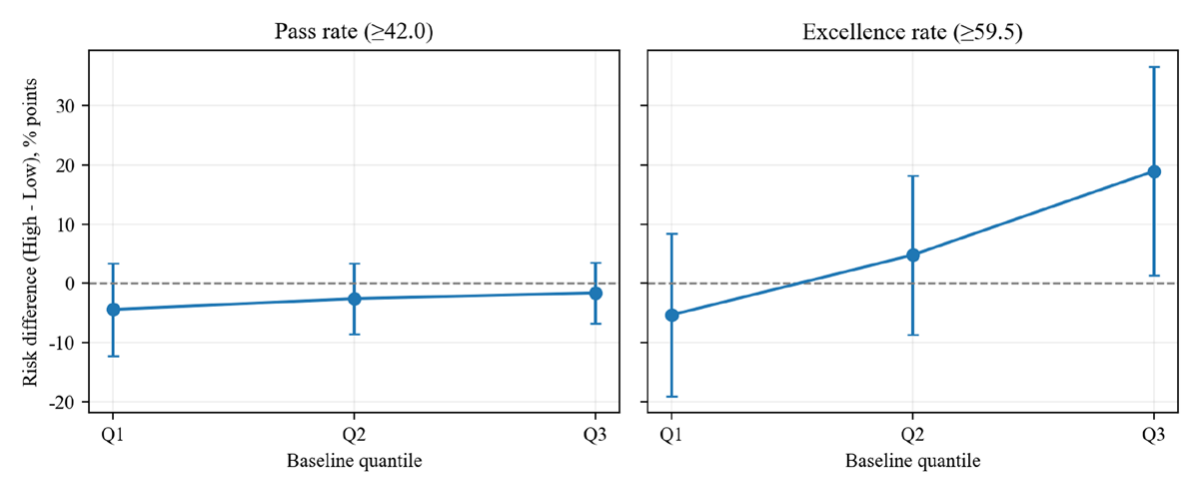
Risk difference by baseline ability quantile: pass (≥42) vs excellence (≥59.5). For pass rate, all point estimates were negative with CIs crossing zero, indicating no clear differences across baseline ability. For excellence rate, the Q1 estimate was negative and imprecise (CI crossed zero), whereas the Q3 estimate was positive and significant (CI excluded zero); sample sizes by baseline ability quartile were (1) for Q1: n=40 (low-intensity=21, high-intensity=19), (2) for Q2: n=78 (low-intensity=33, high-intensity=45), and (3) for Q3: n=39 (low-intensity=20, high-intensity=19).

Marginal effects suggested that higher-intensity practice was associated with larger benefits among students with higher baseline ability (Q3), including higher final examination scores (marginal effect +2.88 points, 95% CI 0.39-5.37; *P*=.02) and a higher probability of excellence (marginal effect +18.93 percentage points, 95% CI 1.32-36.54; *P*=.03; Table 5; Figure 5; Figure 6). At lower baseline ability quantiles (Q1-Q2), marginal effects were not statistically significant for any outcome. Pass rate showed no differences across quantiles, with both clusters maintaining high performance (>94%; Table 5; Figure 6). In summary, these exploratory findings suggested that higher practice intensity may be more beneficial for academically stronger students.

**Table 5:** Marginal effects of high vs low practice intensity by baseline ability quantile among matched artificial intelligence–powered medical history-taking training and evaluation system users (n=157).

Outcome and baseline quantile^a^		Baseline *z* score	Marginal effect (95% CI)	*P* value
**Final score (points)**				
	Q1	−0.45	0.39 (−1.98 to 2.75)	.75
	Q2	0.07	1.72 (−0.29 to 3.73)	.09
	Q3	0.52	2.88 (0.39 to 5.37)	.02
**Excellence rate (percentage points)**				
	Q1	−0.45	−5.32 (−19.04 to 8.40)	.45
	Q2	0.07	4.79 (−8.62 to 18.21)	.48
	Q3	0.52	18.93 (1.32 to 36.54)	.04
**Pass rate (percentage points)**				
	Q1	−0.45	−4.4 (−12.21 to 3.41)	.27
	Q2	0.07	−2.58 (−8.50 to 3.35)	.39
	Q3	0.52	−1.59 (−6.72 to 3.53)	.54

^a^Q1, Q2, and Q3 represent baseline ability at 25th, 50th, and 75th percentiles, respectively.

## Discussion

### Principal Findings

This retrospective cohort study provides real-world evidence on the educational effectiveness of AI-supported medical history-taking training in routine curricula and offers insights relevant to instructional design and precision-oriented medical education. In propensity score–matched analyses, voluntary extracurricular AMTES use was associated with significantly higher scores on the final high-stakes history-taking examination compared with matched nonusers. Among users, digital trace-based clustering identified distinct low- and high-intensity practice patterns; however, more intensive engagement was not consistently associated with additional performance gains. Exploratory ATI analyses further suggested that baseline academic performance may moderate the incremental benefit of intensive engagement, indicating potential heterogeneity in AI-supported skills training.

### Bridging the Gap: Real-World Effectiveness and Practical Value in a High-Baseline Context

This study’s primary contribution is bridging the critical gap between the validated technical feasibility of an AI educational tool and its real-world educational effectiveness. Building upon our previous work that established the AMTES as a technically reliable system [15], this study addressed a more consequential question, that is, does voluntary use of this tool translate into tangible learning gains in an authentic educational context?

Through rigorous PSM, which aligns with recommended practices for causal inference in education [39,40], we observed a statistically and educationally meaningful improvement in final practical skills examination scores among AMTES users compared with matched nonusers. Sensitivity analyses suggested that this finding was robust to unobserved confounding of weak strength.

To our knowledge, this study is among the first to provide empirical evidence that AI-driven history-taking training can yield measurable improvements in high-stakes examination outcomes. Previous studies in this domain have largely focused on system feasibility [20,41]. Although few studies have begun to examine educational effectiveness, such evaluations have typically been conducted under tightly controlled experimental conditions. For instance, in a recent randomized controlled trial, Wang et al [23] compared GPT-based VPs with traditional teacher-facilitated role-playing and reported substantial improvements in structured history-taking scores (Cohen *d*=2.6 approximately) [23]. Our study extends this line of research by demonstrating the effectiveness of AMTES when voluntarily integrated into the routine curriculum, thereby achieving high ecological validity. Moreover, the training cases were intentionally designed to differ from the final examination case in both organ system and core symptoms, ruling out rote memorization or a narrow “teaching to the test” as plausible explanations. Instead, the observed gains are most consistent with a genuine enhancement of students’ generalizable history-taking competence, a fundamental and transferable clinical skill that supports diagnostic reasoning and doctor-patient communication [5,41].

Crucially, the significance of this finding is amplified by the study’s context. Before accessing AMTES, all students had already received a high-quality curriculum that included in-person SP training and direct feedback from clinical instructors. In this setting, demonstrating an incremental benefit of 2.09 points (approximately 3% of the 70-point total) is considerably more challenging than improving performance from a novice baseline. This supports previous findings that AI tools can still provide meaningful gains even within a high-quality, resource-intensive educational framework [27].

In practical terms, this improvement was achieved with favorable cost-effectiveness. AMTES functioned as a low-cost, scalable tool that provided flexible, on-demand practice without increasing faculty workload or consuming scheduled class time [15]. Given the negligible marginal cost of providing additional practice, the effect size represents a substantial educational return on investment. This is consistent with previous work showing that AI-driven virtual SPs can serve as a cost-efficient supplement to traditional SP training [42], reducing personnel and logistical costs while providing scalable and flexible practice opportunities that can be integrated seamlessly into the curriculum.

### Unveiling Authentic SRL Through Digital Traces

Our analyses of AMTES usage logs for the second research question revealed 2 distinct practice profiles among users—a low-intensity group and a high-intensity group. Notably, these clusters differed not only in frequency of use but also in case coverage and interaction depth, suggesting combined differences in practice quantity and quality. Although the 2 groups differed in practice behaviors, these pronounced engagement differences did not translate into statistically significant differences in final practical skills examination scores. This finding warrants cautious interpretation, as the absence of statistical significance should not be construed as evidence that practice volume or depth of engagement is unimportant for skill acquisition. Rather, it suggests that the effectiveness of AMTES may not follow a straightforward dose-response relationship within the observed range of practice behaviors in this cohort.

SRL offers a useful framework for interpreting such practice patterns [24,26]. Rather than relying on self-reported measures, which may not capture SRL as it unfolds over time [25], we used objective process data from AMTES to characterize how students actually engaged with history-taking practice. Digital traces, such as practice frequency, temporal distribution, case coverage, repetition rate, and dialogue length, provided behavioral indicators of how learners planned, monitored, and adjusted their use of the system over time, yielding a higher-fidelity picture of their engagement in this educational context. The clustering approach enabled a more fine-grained description of SRL-related engagement patterns than a simple user-nonuser distinction.

The absence of a clear performance advantage for the high-intensity cluster is also consistent with previous work indicating that relationships between SRL and academic achievement are not uniformly strong or statistically significant [24]. Although several studies have reported positive links between SRL and exam performance or clinical skills [43-45], other longitudinal and clinical training studies have reported weak or inconsistent associations between specific SRL components and achievement outcomes, with some strategies showing no significant relationships [25,46]. In our setting, this may help explain why greater AMTES use did not yield markedly higher scores. AMTES provided structured opportunities for reflective, feedback-driven, deliberate practice [15]. Students who actively processed feedback and adapted their questioning strategies were likely able to translate even modest amounts of practice into meaningful gains, whereas undirected, repetitive sessions produced diminishing returns and limited additional benefit. This interpretation aligns with contemporary SRL theory, which emphasizes metacognitive monitoring, strategic planning, and iterative adjustment as core drivers of skill acquisition [26,47]. Additionally, opportunity costs may partly explain this pattern, as repeated practice within a small set of validated cases could displace other high-yield learning activities (eg, advanced case discussions or authentic clinical encounters) [48].

### Heterogeneity of Effect: Exploratory ATI Patterns

In authentic, opt-in implementations, average dose-response relationships may appear weak or inconsistent when effects are heterogeneous and potentially nonlinear across learners [30]. Therefore, the nonsignificant difference between practice intensity groups does not preclude differential effects across learner subgroups—benefits for higher-ability students, alongside smaller or null effects for lower-ability students, could aggregate to a near-zero average difference. To examine this possibility, we conducted an exploratory analysis guided by the ATI framework [49].

Our exploratory ATI analysis examined whether the benefits of intensive AMTES engagement varied by baseline academic ability. Among AMTES users, regression models suggested that the added value of high- versus low-intensity engagement may be moderated by baseline academic ability. The pattern appeared more evident for excellence-related outcomes and among higher-performing students, whereas pass rates were uniformly high with limited room for differentiation.

These heterogeneity findings should be interpreted as hypothesis-generating. However, the observed pattern is conceptually consistent with the ATI framework [28,30,49], which posits that the effects of an instructional treatment may depend systematically on learner characteristics. One possible explanation is that cognitive resources and instructional fit may shape how effectively learners act on structured feedback [49], such that stronger baseline competence may support greater gains [47]. Conversely, learners with weaker foundations may require additional instructional scaffolding to benefit fully from intensive practice [50].

Overall, these exploratory findings highlight the potential value of aligning AI-supported practice design (eg, case diversity, adaptive difficulty, and feedback scaffolding) with learners’ baseline competence to optimize educational impact and instructional fit [48]. These considerations inform the practical implications discussed next, including design and instructional strategies for personalized, adaptive use of AMTES within routine curricula.

### Implications for Teaching Practice: Toward SRL-Supportive and Precision Medical Education

Our findings suggest that AI systems, such as AMTES, should be evaluated not only as technical innovations, but also by their impact in authentic teaching contexts. When integrated into modern medical curricula, such tools need to be used in a learner-oriented way that supports SRL [51] and precision education [52].

From an SRL perspective, AMTES is most valuable when it supports students in planning, monitoring, and adjusting their learning [26]. As Lajoie and Gube [51] argued, technology-enhanced learning environments should scaffold SRL processes, such as planning, monitoring, and reflection, to cultivate adaptive expertise rather than being used solely for automated, routine practice. Instructors can therefore help students establish efficient usage patterns grounded in SRL by deliberately structuring AMTES practice around reflection and feedback integration. This approach may encourage more purposeful, feedback-informed practice.

At the same time, our exploratory findings suggest that AMTES may support learners across ability levels, but intensive engagement may yield greater gains among students with stronger baseline competence. This pattern aligns with the emerging framework of precision medical education, which emphasizes leveraging longitudinal learning analytics and data-driven (including AI-enabled) insights to inform personalized support and interventions tailored to individual learner needs [52]. Instructors may therefore support students by promoting efficient, SRL-aligned engagement strategies and encouraging practice plans that are responsive to individual learning needs.

Taken together, these implications point to a learner-centered approach to integrating AMTES into medical curricula. In this way, AI-based systems, such as AMTES, are positioned not as simple extra practice tools, but as supports for self-regulation and individualized learning trajectories that can complement existing instruction.

### Limitations and Future Directions

Several limitations must be acknowledged. First, as an observational retrospective cohort study, residual confounding cannot be fully ruled out [53]. The Rosenbaum bounds sensitivity analysis indicated robustness only to unmeasured confounding of weak strength, and unobserved factors, such as intrinsic motivation, SRL skills [54], digital literacy [55], or time management could, in principle, still influence both AMTES use and examination performance. However, our matching model was based on a comprehensive set of covariates, including 2 demographic variables (age and sex) and 3 key baseline academic performance metrics (midterm, previous theory, and practical examination scores) collected before the AMTES usage period. These performance metrics are widely recognized as strong proxies for underlying traits, such as motivation, self-discipline, and study habits [34,35,56,57]. By adjusting for these performance indicators, we likely reduced a substantial proportion of confounding related to these latent characteristics, although the possibility of residual bias from other unmeasured factors cannot be completely excluded.

Second, the clustering of practice patterns and the interaction analysis were exploratory in nature. Interaction effects are inherently difficult to estimate precisely, and our heterogeneity analyses should be treated as exploratory and hypothesis-generating [29,30]. Future studies with larger samples, richer case libraries, and longer follow-up periods are warranted to further examine whether practice patterns yield differential benefits across learners with different baseline competencies.

Finally, this study was conducted at a single institution over 1 academic cycle, and it only assessed immediate examination outcomes, lacking follow-up on long-term effects. It remains unclear whether AMTES training can lead to long-term improvements in students’ performance during clinical clerkships or subsequent examinations, or its deeper impact on motivation and SRL habits. Furthermore, the differential effects of varying numbers of training cases and the system’s applicability across different cultures and educational systems remain to be validated. Generalizing the findings to other institutions and teaching environments requires caution.

Future research should use larger-scale designs (such as randomized controlled trials or expanded real-world studies), include follow-up on subsequent student performance and attitudinal changes, and extend validation to different grade levels, courses, and multiple centers. Such work will be important for confirming the robustness of the present findings and for refining how AI-based history-taking trainers like AMTES can best be integrated into medical curricula worldwide.

### Conclusion

Building upon previous technical validation, this study contributes real-world evidence from an authentic, high-baseline educational context in which AMTES was offered as a voluntary extracurricular supplement alongside the routine curriculum. Compared with many previous works focusing on technical feasibility or short-term controlled trials, this study found voluntary extracurricular AMTES use was associated with modest yet meaningful improvements in summative history-taking performance. Exploratory analyses suggested that the added value of more intensive engagement may be moderated by baseline academic ability, indicating that the benefits of AI-supported practice could vary across learner profiles. These findings support the scalability and resource efficiency of AI-enabled supplementary training and inform precision-oriented instructional design. Future studies should prospectively evaluate heterogeneous effects across diverse learners and test adaptive implementation strategies, including expanded case libraries, SRL-guided scaffolding, and ability-aligned progression when implementing AI-based training tools.

## Appendix

Multimedia Appendix 1 Normality test for within-pair differences in final practical skills examination scores.

Multimedia Appendix 2 Clustering features construction and selection.

Multimedia Appendix 3 Determination of the optimal number of clusters (K).

Multimedia Appendix 4 Propensity score distributions before and after matching.

## Abbreviations

AI: artificial intelligence
AMTES: AI-powered Medical History-Taking Training and Evaluation System
ATI: aptitude-treatment interaction
ATT: average treatment effect on the Treated
LLM: large language model
PSM: propensity score matching
SMD: standardized mean difference
SP: standardized patient
SRL: self-regulated learning
VP: virtual patient

## Data Availability

The datasets generated or analyzed during this study are available from the corresponding author on reasonable request.
