# Supplementary material for: Real-World Impact and Educational Effectiveness of an AI-Powered Medical History-Taking System: Retrospective Propensity Score-Matched Cohort Study
Source: JMIR Med Educ. 2026 Feb 24;12:e89367. doi: 10.2196/89367 (PMC12976603; doi:10.2196/89367)
Supplement: Multimedia Appendix 1 [file mededu_v12i1e89367_app1.pdf]

Multimedia Appendix 1: Normality test for within-pair differences in final examination scores.

Table 1. Shapiro－Wilk test for within-pair differences in final examination scores.

Statistic (W)	P value	Number of pairs
0.992	.582	157

We assessed the normality assumption for the within-pair differences in final examination scores (treated − control) using the Shapiro–Wilk test. The results indicated no evidence against normality, supporting the use of paired *t* tests to estimate the average treatment effect on the treated (ATT).
