# Supplementary material for: Real-World Impact and Educational Effectiveness of an AI-Powered Medical History-Taking System: Retrospective Propensity Score-Matched Cohort Study
Source: JMIR Med Educ. 2026 Feb 24;12:e89367. doi: 10.2196/89367 (PMC12976603; doi:10.2196/89367)
Supplement: Multimedia Appendix 2 [file mededu_v12i1e89367_app2.pdf]

Multimedia Appendix 2: Clustering features construction and selection

Table 1. Log-derived features exacted before clustering analysis.[a-e]

Dimension	Feature[a]	Interpretation
Engagement & Coverage	Total_sessions	Total number of AMTES sessions completed
	Unique_cases	Number of distinct cases practiced
Practice Strategy	Repeat_rate[b]	Proportion of repeated practice
	Case_entropy	Shannon entropy of case selection
Outcome Quality	Avg_score	Mean session score across all sessions
	Min_score	Lowest AMTES session score
	Max_score	Highest AMTES session score
Interaction Dynamics	Avg_turns[c]	Average number of dialogue turns per session
	Min_turns[d]	Minimum number of dialogue turns in any session
	Max_turns	Maximum number of dialogue turns in any session
	Turns_per_min[e]	Overall dialogue turns per minute

[a]All features were standardized (z scores) before clustering

[b]Repeat_rate was calculated as 1 - unique_cases/sessions_total.

[c,d,e]Skewed variables were transformed using a log1p transformation (log[x + 1]).

Before clustering, we extracted eleven features from student practice logs across four conceptual dimensions. Their definitions and preprocessing steps are summarized in Table 1.

Figure 1. Spearman correlation heatmap

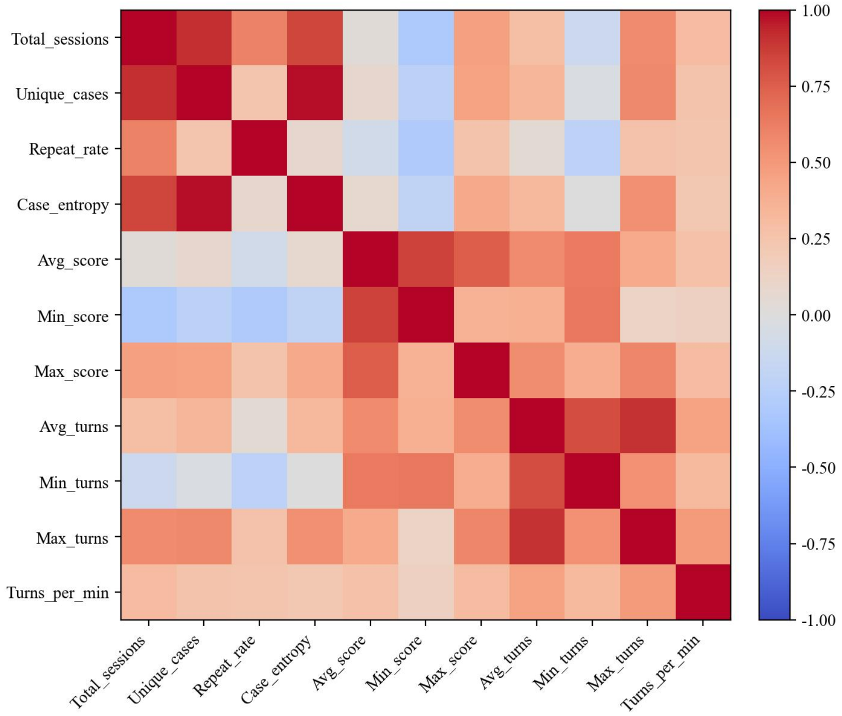

The Spearman correlation heatmap of the log-derived training features showed clear clusters of interrelated variables, indicating substantial redundancy among several candidate indicators. In particular, engagement/coverage metrics and practice stategy metrics (e.g., total sessions and case-related indices) tended to co-vary, and summary statistics derived from the same underlying construct (e.g., mean vs. minimum/maximum) exhibited strong concordance. Overall, the heatmap supported the need for correlation-based feature reduction before clustering to avoid overweighting highly overlapping information.

Table 2. Highly correlated candidate feature pairs (Spearman $|\rho| \geq 0.80$).

Feature 1	Feature 2	Spearman $|\rho|$
Unique_cases	Case_entropy	0.980
Total_sessions	Unique_cases	0.913
Avg_turns	Max_turns	0.9
Avg_score	Min_score	0.856
Total_sessions	Case_entropy	0.837
Avg_turns	Turns_per_min	0.816

Consistent with the heatmap, Spearman correlation screening revealed substantial redundancy ($|\rho| \geqslant 0.8$) among several candidate features: average session score was strongly correlated with minimum score, average dialogue turns was strongly correlated with minimum/maximum turns, and case entropy was highly correlated with the number of unique cases. To avoid over-weighting closely related indicators in

the clustering, we reduced the feature set by retaining a single representative from each highly correlated group while preserving conceptual coverage. The final five clustering features were total sessions (log1p-transformed) and number of unique cases (engagement and coverage), average session score (platform performance), average dialogue turns per session (interaction volume), and turns per minute (interaction pace). Although total sessions and unique cases were also highly correlated ($\rho \approx 0.91$), we retained both because they capture complementary behavioral constructs (practice intensity vs breadth of coverage).

Table 3. Multicollinearity diagnostics for retained clustering features.

Retained feature	VIF	R^2 from auxiliary regression[a]
Total_sessions	4.897	0.796
Unique_cases	4.93	0.797
Avg_score	1.654	0.395
Avg_turns	2.026	0.506
Turns_per_min	1.369	0.269

[a]For each feature, R^2 is obtained by regressing that feature on the remaining retained features; VIF = $1/(1-R^2)$

To further assess multicollinearity among the retained five features, we computed variance inflation factors (VIFs) as an auxiliary check. The largest VIFs were observed for total_sessions (log1p-transformed) and unique_cases (both ~4.9), while the remaining features had lower VIFs (approximately 1.4–2.0). These values are below common concern thresholds (e.g., VIF>5, suggesting that the final five-feature set does not exhibit severe multicollinearity and is suitable for clustering without excessive redundancy.
