# Supplementary material for: Real-World Impact and Educational Effectiveness of an AI-Powered Medical History-Taking System: Retrospective Propensity Score-Matched Cohort Study
Source: JMIR Med Educ. 2026 Feb 24;12:e89367. doi: 10.2196/89367 (PMC12976603; doi:10.2196/89367)
Supplement: Multimedia Appendix 3 [file mededu_v12i1e89367_app3.pdf]

Multimedia Appendix 3: Determination of the optimal number of clusters (K).

To determine an appropriate number of clusters (K) for the K-means analysis of AMTES practice behavior, we evaluated candidate solutions with different K values (2–8) using silhouette coefficients.

The silhouette analysis showed that K=2 achieved the highest average silhouette score among K=2–8 (0.316), with a monotonic decline for larger K values and only minor rebounds (Table 1; Figure 1).

Table 1. Silhouette scores for candidate K values (2–8)

K value	Silhouette
2	0.316
3	0.297
4	0.269
5	0.258
6	0.253
7	0.256
8	0.259

Figure 1. Silhouette plot

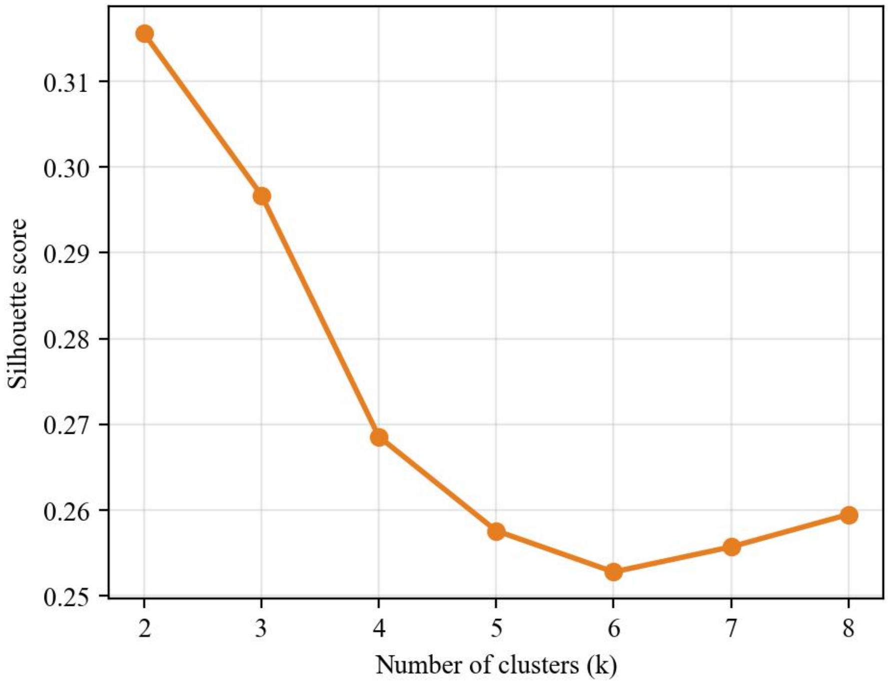

Table 2. Bootstrap selection frequencies for the number of clusters (K) under silhouette criteria (n=500 resamples)

Number of clusters (K)	Count (n)	Percentage (%)
2	257	51.4
3	114	22.8
4	12	2.4
5	2	0.4
6	4	0.8
7	12	2.4
8	99	19.8

In bootstrap resampling (n=500), K=2 was the most frequently selected solution under the silhouette criterion (51.4%), followed by K=3 (22.8%) and K=8 (19.8%) (Table 2).

Given the higher silhouette score at K=2 in the full sample and the interpretability of a two-profile solution, we selected K=2 for the primary clustering analysis.
