# Supplementary material for: Real-World Impact and Educational Effectiveness of an AI-Powered Medical History-Taking System: Retrospective Propensity Score-Matched Cohort Study
Source: JMIR Med Educ. 2026 Feb 24;12:e89367. doi: 10.2196/89367 (PMC12976603; doi:10.2196/89367)
Supplement: Multimedia Appendix 4 [file mededu_v12i1e89367_app4.pdf]

Multimedia Appendix 4: Propensity score distributions before and after matching.

Figure 1. Propensity score distributions before matching.

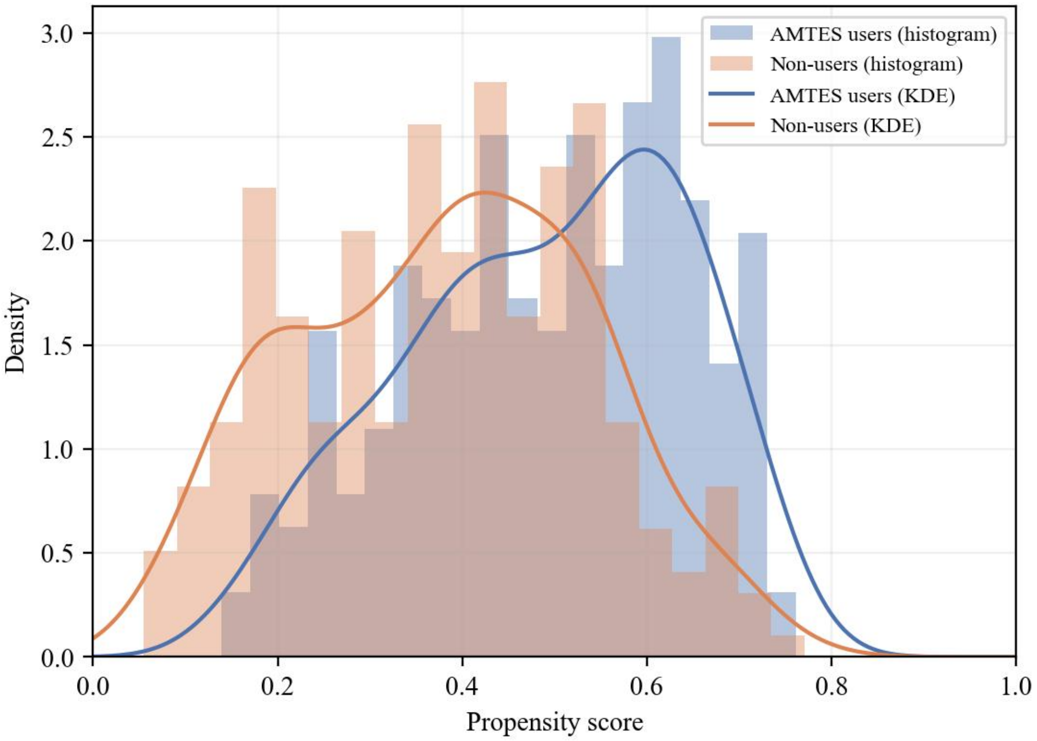

Figure 2. Propensity score distributions after matching.

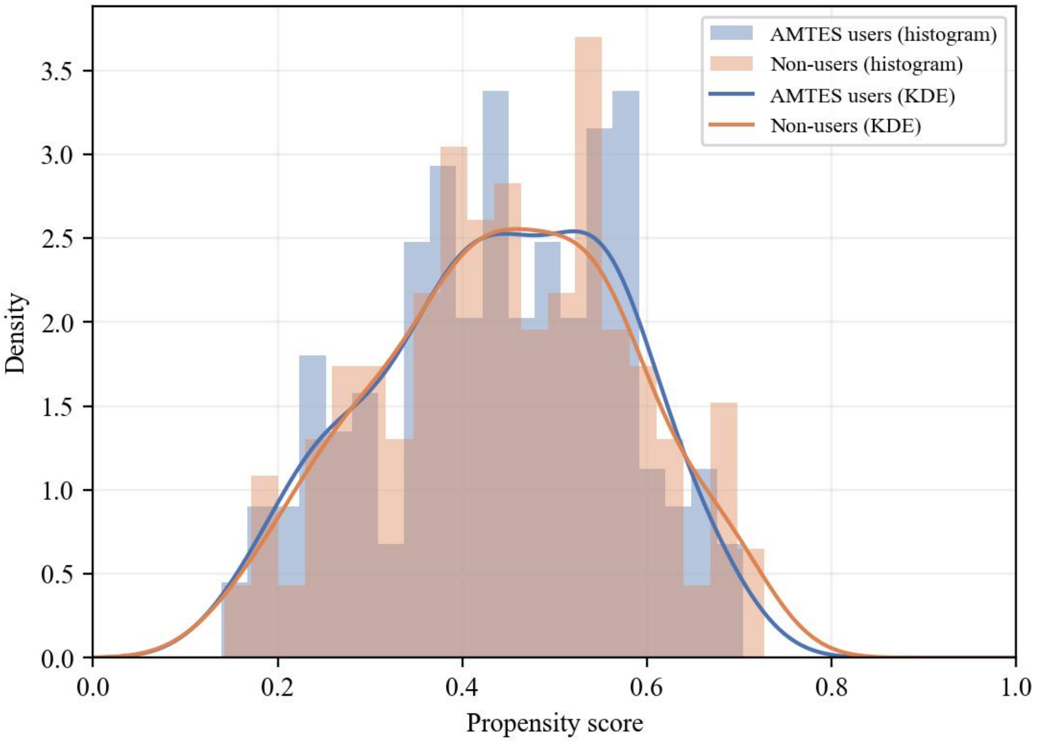

This appendix displays the distribution of propensity scores for AMTES users and non-users before and after matching. Figure 1 shows substantial separation between groups prior to matching, whereas Figure 2 shows largely overlapping distributions after 1:1 nearest-neighbor matching with a caliper of 0.2 SD of the logit of the propensity score. These plots complement the standardized mean differences and AUC values reported in the main text and support the adequacy of covariate balance in the matched sample.
